# Retroviral Integration Site Selection

**DOI:** 10.3390/v2010111

**Published:** 2010-01-12

**Authors:** Sébastien Desfarges, Angela Ciuffi

**Affiliations:** Institute of Microbiology, University Hospital Center and University of Lausanne, Bugnon 48, CH-1011 Lausanne, Switzerland; E-Mail: Sebastien.Desfarges@chuv.ch

**Keywords:** retrovirus, integration, site selection, integrase, HIV, LEDGF, LEDGF/p75, PSIP1, lentivirus, transcription

## Abstract

The stable insertion of a copy of their genome into the host cell genome is an essential step of the life cycle of retroviruses. The site of viral DNA integration, mediated by the viral-encoded integrase enzyme, has important consequences for both the virus and the host cell. The analysis of retroviral integration site distribution was facilitated by the availability of the human genome sequence, revealing the non-random feature of integration site selection and identifying different favored and disfavored genomic locations for individual retroviruses. This review will summarize the current knowledge about retroviral differences in their integration site preferences as well as the mechanisms involved in this process.

## 1. Introduction

**Figure 1.**
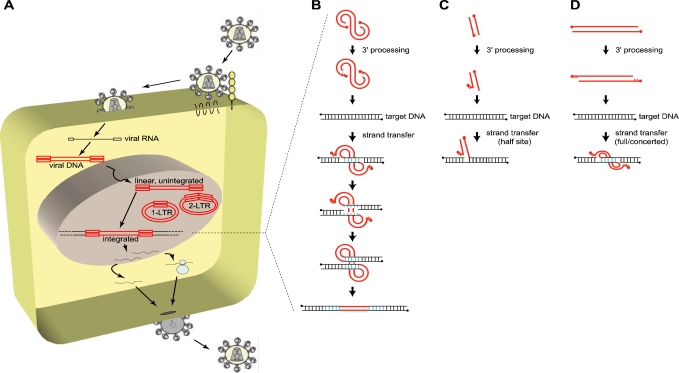
Overview of the early steps of HIV-1 life cycle. **(A)** To enter a target cell, HIV-1 gp120 binds to specific cellular receptors, *i.e.,* CD4 and a chemokine coreceptor (CCR5 or CXCR4), triggering the gp41-mediated fusion between the viral and the cellular membrane, and releasing the viral core in the cytoplasm of the host cell. The viral single stranded, positive, RNA genome (black line, flanked by open black squares depicting R-U5 and U3-R in its 5′ and 3′ termini respectively) is reverse transcribed into a linear double stranded cDNA copy (red line, flanked by open red squares representing the LTR = U3-R-U5), which is a component of the preintegration complex (PIC), also containing the viral integrase (IN), as well as other viral and cellular proteins. The PIC is translocated to the nucleus and the viral cDNA is either integrated through the action of IN or remains unintegrated (linear, 1-LTR circles, 2-LTR circles). From this point on, the cellular machinery of the host is recruited to transcribe the viral genome in order to produce all the components required to generate newly infectious particles. **(B)** The integration process is divided into three major steps: the 3′ processing and the strand transfer reaction, both catalyzed by IN, and the repair of the integrated viral DNA by the DNA repair machinery of the host cell. The PIC-containing viral DNA (red line, with 5′ ends depicted by filled circles) is first processed by the IN-mediated removal of a dinucleotide (GT) at each 3′ end of the viral DNA, leaving a protruding (AC) dinucleotide at the 5′ ends. IN then catalyzes the stable insertion of the processed viral DNA into a target DNA (black line), by simultaneously and asymmetrically breaking the target DNA 5 bp apart (blue bonds) (4 to 6 bp depending on the retrovirus) and joining it to the 3′ recessed ends of the viral DNA, leaving an integration intermediate with unpaired bases at each viral-target DNA junction. The DNA repair machinery of the host cell fills in the five nucleotide gap at each side of the viral DNA and removes the two 5′ overhang nucleotides from the viral DNA, resulting in the duplication of 5 bp of the target DNA at both sides of the proviral DNA. (C and D) Schematic concepts of *in vitro* integration assays showing half-site integration **(C)** and concerted or full-site integration **(D)**.

The principal feature of retroviruses is that upon entry and release of their viral RNA genome into the cytoplasm of the host cell, it is reverse transcribed by the viral reverse transcriptase into a linear double stranded cDNA copy (Figure 1A) (for reviews, see [1–4]). This viral DNA is not naked, but is associated with viral and cellular proteins in a nucleoprotein complex called the preintegration complex (PIC). Depending on the retrovirus, the PIC is subsequently translocated into the nucleus, either actively through nuclear pores, or upon nuclear membrane disruption occurring during mitosis. There, it is, either integrated into the genome of the host cell, or remains unintegrated for a certain time, or is degraded (Figure 1) (for reviews, see [3,5]).

The insertion of the viral DNA into the host cell genome is catalyzed by the virally encoded integrase (IN) enzyme (for reviews, see [6–9]). Retroviral INs typically range between 280 and 450 amino acids (HIV-1 IN: 288 amino acids, 32 kDa), and are characterized by three functional domains: (i) the N-terminal domain, containing an HHCC zinc-binding motif, (ii) the catalytic core domain (residues 50-212 of HIV-1), containing the critical magnesium-binding D-D-35-E motif that constitutes the active site, and (iii) the C-terminal domain. The three domains of IN appear to be involved in DNA binding and multimerization. Indeed, the full concerted integration seems to require an IN tetramer, *i.e.,* one IN dimer at each viral end [10–14].

Although unintegrated viral DNA can be used as template for viral transcription [15–17], integration is required for productive viral replication. However, the efficiency of integration is quite low, and depends on restrictions occurring during the early steps of infection. By infecting human osteosarcoma (HOS) cells with VSV-G pseudotyped HIV particles, Thomas *et al.* measured that only 5% of HIV viruses successfully entered the cell and initiated reverse transcription [18]. Of these, 28% (*i.e.,* 1.5% total) completed reverse transcription, translocated to the nucleus (with an efficiency ranging around 2–3% according to data using IN-eGFP fusion proteins from Cereseto and collaborators [19,20]) and finally only ∼13% of viruses that initiated reverse transcription achieved insertion in the host cell genome, which represented 0.41% of the virus input [18].

The site of viral DNA insertion is critical for the virus, as it can influence the rate of viral transcription. Indeed, integration into transcriptionally active regions may favor viral gene expression, thus facilitating productive infectious progeny particles, while integration into transcriptionally repressed chromatin may disfavor viral gene expression, thus possibly facilitating viral latency [21–24].

The ability of retroviruses to integrate has also important consequences for the host as it can affect the expression of genes surrounding the proviral DNA. Indeed, viral DNA disruptive insertion into a gene may alter its expression (reduced gene expression), thereby potentially affecting cellular physiology. More importantly, the activity of inserted viral promoters or enhancers near cellular genes may also affect their physiological expression (increased gene expression), potentially leading to tumorigenesis when these genes are proto-oncogenes [25,26]. This process, known as insertional mutagenesis, raised justified critical issues regarding the safety of retroviral-based vectors used in gene therapy (for reviews, see [27–32]). However, not all retroviruses display the same genotoxic potential, as gammaretroviruses for example appeared to be more prone to insertional mutagenesis than lentiviruses [33–37]. This phenomenon can be partly explained by their preferred genomic site for proviral DNA insertion, *i.e.,* into promoter regions for gammaretroviruses and along transcription units for lentiviruses.

It is now clear that the chromosomal site of viral DNA integration is not random, but in contrast that retroviruses display specific preferences at distinct genomic positions. This review will summarize the current knowledge about retrovirus-specific favored integration sites, as well as the current models explaining these preferences.

## 2. Integration targeting *in vitro*

*In vitro*, IN is sufficient to carry out the first two steps of the integration reaction, *i.e.,* 3′ processing and strand transfer reactions, resulting in the covalent attachment of the viral DNA on virtually any DNA target (random integration) (Figure 1B) [38]. To succeed, three principal components are minimally required: (i) purified viral integrase, (ii) a donor DNA mimicking a viral DNA terminal sequence to be recognized by IN, and (iii) an acceptor DNA in which the donor DNA will be inserted (for more details, see [7]).

In the first *in vitro* assays, the donor DNA consisted in short oligonucleotide duplexes (21 bp minimum) containing the terminal LTR sequence (either U3 or U5), allowing to reproduce the 3′ processing efficiently, as well as the strand transfer reaction (albeit with lower efficiency). However, these were only half-site integration as only one donor DNA (e.g. one viral LTR) was inserted in the acceptor DNA (Figure 1C) [39], and not both in a concerted motion. This gave rise to the development of new assays, full-site or concerted integration assays, which use a longer donor DNA containing both terminal sequences (Figure 1D) [40,41].

These studies showed that *in vitro*, HIV IN displayed only a weak preference for the primary DNA sequence [42–49], slightly favoring the palindromic TNN**GT(A/T)AC**NNA DNA sequence (bold nucleotides indicate the asymmetrical insertion points, resulting in the final 5 bp duplication flanking the proviral DNA, depicted in blue in Figure 1B). Furthermore, the addition of nucleosomes on the target DNA improved the *in vitro* efficiency of integration, and favored integration on distorted DNA and outwardly-facing major grooves sites of the nucleosomal DNA [44,46,47,50–53].

In order to investigate whether simple tethering of IN to a specific DNA site could confer integration preferences *in vitro*, fusions of IN to specific DNA binding proteins were engineered [54]. The fusion of HIV IN to the DNA binding domain of λ repressor (λR) lead to increased integration targeting at sites surrounding the predetermined λR DNA binding sites (λ operator sites) [54]. Fusion of IN with other DNA binding proteins such as LexA [55] or the polydactyl Zinc finger protein E2C [56] reached similar results. These studies provided proof-of-concept that integration site selection *in vitro* could be modified and redirected more preferentially to specific DNA sites.

## 3. Integration targeting *in vivo*

The availability of the human genome sequence and other vertebrate genomes made possible to interrogate where in the host cell genome retroviruses integrated, and more precisely what were the chromosomal features (according to current genomic annotations) that were favored for retroviral integration. To achieve this, host DNA regions flanking the proviral DNAs were amplified, sequenced, and finally aligned to the host genome sequence (method overview reviewed in [32,57,58]).

Schroder *et al.*, in 2002, revealed for the first time that HIV favored integration in transcription units and disfavored Alu repeats [59]. One year later, Wu *et al.* showed that murine leukemia virus (MLV) had distinct preferences, favoring integration at transcription start sites and CpG islands [60]. Since then, multiple genome-wide studies confirmed these preferences and revealed the integration site preferences for almost all retroviral genera, with the exception of epsilonretroviruses (Tables 1 and 2).

These studies demonstrated that *in vivo* the site of retroviral integration was not random, and that integration site preferences were retrovirus-specific (Table 2): lentiviruses favor integration in active transcription units, with no preference along the transcript, nor for introns or exons; gammaretroviruses, spumaviruses and endogenous retroviruses (HERV class II) integrate preferentially around transcription start sites and CpG islands, features associated with host gene promoters; alpharetroviruses and deltaretroviruses displayed only weak preferences for integrating in transcription units and CpG islands; and finally betaretroviruses show no integration site preferences, displaying a random distribution of integration sites in the host genome.

The integration site preferences are not host-specific as the same distribution of integration sites can be observed in different host vertebrate cells, including human, simian, murine, avian and canine cells (Table 1). Furthermore, integration targeting is independent of the route of viral entry, as HIV-based vectors using a natural CCR5-tropic HIV envelope or a VSV-G pseudotype envelope displayed the same integration site distribution [67].

Three models, which are not mutually exclusive, have been proposed to date to explain integration site selection: (i) chromatin accessibility, (ii) cell cycle effects, and (iii) tethering mechanism. However, while the first two models can globally influence integration site targeting, only the last one - integration by a tethering mechanism - can provide a logical explanation to the observed differences of integration targeting preferences among retroviruses.

### 3.1. The chromatin accessibility model

According to this model, the structure of the chromatin, either relaxed or condensed, may influence the accessibility of target DNA sequences to preintegration complexes, thereby affecting integration.

*In vivo*, retroviral integration displayed a weak preference for the primary DNA sequence, similar to the one observed *in vitro* [60,69,70,72,90,94,96–99]. Furthermore, HIV integration *in vivo* also favors major grooves facing outwards from the nucleosome core, as predicted by nucleosome positioning [72,100]. These data indicated that local chromatin structure, such as A/T-rich distorted DNA and outwardly-facing major grooves of the nucleosomal DNA, may facilitate integration, however this cannot fully explain the observed differences in retroviral integration site distribution.

The retroviral differences in favored integration target site selection observed *in vivo* argued against chromatin accessibility being the principal determinant explaining integration targeting (Table 2). Indeed, if the accessibility of chromatin was the key player, all retroviruses would display the same integration site distribution pattern, favoring reachable chromatin.

Additional evidence against this model playing a major role in integration targeting came from the correlation analysis of mapped HIV and MLV integration sites with mapped DNase I hypersensitive sites. DNase I cleavage sites are used as a surrogate marker for accessible chromatin, and are enriched in the 5′ ends of transcription units and CpG islands [66,101]. This study revealed that MLV integrated preferentially in 2-kb intervals surrounding DNase I hypersensitive sites, compatible with favored MLV integration sites in promoter regions. In contrast, HIV integration did not display such a preference, consistent with favored integration in transcription units and not promoter regions. Therefore, although chromatin accessibility may influence MLV integration site preferences, it does not seem to affect significantly HIV integration site distribution.

In conclusion, even though chromatin structure can facilitate integration, chromatin accessibility cannot solely explain the differences observed in integration site preferences between HIV and MLV.

### 3.2. The cell cycle model

This model implies that the phase of the cell cycle may influence integration site selection. Indeed, lentiviruses can infect and successfully integrate regardless of the cell cycle stage (dividing or non-dividing) thanks to the active nuclear import of the PIC, while gammaretroviruses can integrate only into dividing cells as they require the disruption of the nuclear membrane occurring during mitosis to contact the host genome. Thus, it is possible that this difference in cell cycling status during viral infection might affect integration site distribution.

To test this hypothesis, HIV integration site distribution was compared between dividing IMR-90 primary lung fibroblasts and non-dividing G1-arrested IMR-90 cells [64]. HIV integration in active transcription units was favored in both dividing and non-dividing cells, with even stronger preferences in non-dividing cells. Similarly, analysis of HIV integration site distribution in non-dividing differentiated human macrophages also revealed a marked preference for transcription units [67,74]. Comparison between quiescent CD4+ T cells and activated CD4+ T cells revealed a similar integration site distribution with favored integration in transcription units and other chromosomal features (gene density, GC-rich regions, DNase I sites), although to a lower extent for resting cells [75,76].

All together, these data argued against a major positive influence of cycling cells in guiding HIV integration in transcription units and cannot explain the integration site selection differences between HIV and MLV.

### 3.3. The tethering protein model

This model implicates that a cellular protein, specific for each retroviral genera, would act as a tethering factor, binding both to specific chromatin sites and to the retroviral preintegration complex.

In principle, any PIC component could serve as the docking point between the PIC and the integration site, thereby dictating the integration target site preferences. PIC candidates include both viral and cellular proteins.

#### 3.3.1. IN and Gag as major viral determinants in integration targeting

To investigate the role of viral PIC components in integration site distribution, Lewinski *et al.* constructed chimeras between HIV and MLV encompassing Gag and IN regions [66]. The transfer of MLV Gag in HIV viruses did not affect the HIV integration site preferences of HIV, suggesting that HIV Gag was not involved in directing HIV integration site selection. The transfer of MLV IN into HIV viruses caused the hybrid to favor integration in transcription start sites and CpG islands, close to MLV phenotype. Addition of MLV Gag and MLV IN in HIV chimeric viruses increased the similarity of target site selection to that of MLV, suggesting that both MLV Gag and IN played a role in determining MLV integration site preferences. To summarize these data indicated that IN is the major viral determinant in shaping specific integration site preferences for HIV and MLV, with an additional, although minor, role of MLV Gag in MLV integration targeting. Recently, Felice *et al.* used a bioinformatic approach to show that in contrast to HIV, MLV integrated preferentially in regions enriched for transcription factor binding sites (as described in the JASPAR database) [73]. Using chimeric constructs for IN and the LTR U3 region between HIV and MLV, they showed that MLV IN was mostly responsible for this targeting, while the MLV U3 region could play a minor role [73].

Recently, Tobaly-Tapiero *et al.* identified a chromatin-binding site in the C-terminus of Gag, essential for PIC binding to host chromosomes, by interacting with H2A/H2B core histones, suggesting that Gag may be a major viral determinant dictating foamy virus (FV) integration site selection, through H2A/H2B tethering [102].

#### 3.3.2. LEDGF/p75 as the major cellular determinant in lentiviral integration targeting

Any cellular protein able to bind both chromatin and HIV IN (the major determinant involved in HIV integration targeting) may represent a candidate tethering factor. Numerous cellular proteins have been proposed, such as IN-interactor 1 (Ini-1), barrier to autointegration factor (BAF), high mobility group A1 (HMGA1), heat shock protein 60 (Hsp60) and lens epithelium-derived growth factor (PSIP1/LEDGF/p75) [2,103–108].

To date, only LEDGF/p75 proved to be a *bona fide* tethering protein, recruiting lentiviral PICs to transcription units, thereby promoting integration efficiency as well as dictating lentiviral integration site selection [32,109–111].

LEDGF/p75 is a 530 amino-acid bimodal protein containing a large N-terminal domain (comprising a PWWP motif, a nuclear localization signal, a dual AT-hook motif and charged regions) responsible for chromatin binding, and a C-terminal domain involved in protein-protein interaction and containing the IN-binding domain [110–112]. LEDGF/p75 is a ubiquitously expressed cellular protein and its cellular role has yet to be fully characterized. Proposed roles for LEDGF/p75 include transcriptional activity function [113], autoantigen in atopic dermatitis and inflammatory conditions [114], and cell survival [115]. Despite an increased perinatal mortality, mice knocked-out for the LEDGF/p75-encoding *psip1* gene survived to adulthood with a range of developmental and neurobehavioral abnormalities, suggesting that LEDGF/p75 is not essential for organism survival [116].

Studies using RNAi to knock-down LEDGF/p75 expression or knock-out murine cells demonstrated that HIV and lentiviruses in general required LEDGF/p75 to efficiently integrate into the host genome [80,117,118]. Indeed, LEDGF/p75-depleted cells revealed a 10-40x decrease in infection efficiency as compared to LEDGF/p75 expressing cells. Furthermore, the analysis of lentiviral (HIV and EIAV) integration site distribution in cells depleted for LEDGF/p75 revealed an altered integration site selection profile, with a decreased preference for transcription units, as well as an increased targeting in transcription start sites and CpG islands, a pattern resembling MLV integration site preferences [63,80,81]. However, LEDGF/p75 did not affect the weak consensus primary DNA sequence favored by lentivirus integration, further suggesting that chromatin structure may facilitate integration more than dictating integration target sites. These data also suggest that, in absence of LEDGF/p75, other tethering proteins might recruit lentiviral PICs, and promote their integration in new specific chromosomal locations.

Consistent with LEDGF/p75 recruiting lentiviral PICs to specific favored integrations, the distribution profile of LEDGF/p75 on host chromosome should parallel HIV integration site distribution profile. In order to investigate this, De Rijck *et al.* used the Dam methylase fused to the N-terminal domain of LEDGF/p75, a technology known as DamID [119]. Sites bound by LEDGF/p75 would induce methylation of proximal adenosine residues in the GATC recognition motif, which can subsequently be identified. DamID analysis of LEDGF/p75 proximal genomic site distribution revealed that LEDGF/p75-binding sites were enriched in genes and poorly present in promoters and intergenic regions, a distribution pattern reminiscent of HIV integration preferences [119]. Preliminary analysis of DNA sequences bound to LEDGF/p75 or LEDGF/p75-containing complex using a chromatin immunoprecipitation-based approach followed by high-throughput sequencing (ChIP-Seq) also revealed a distribution of LEDGF/p75-captured DNA sequences throughout the transcription units, paralleling HIV integration site distribution [120]. Moreover, so far, these studies did not highlight any DNA binding motif specific for LEDGF/p75 [120].

An additional argument for LEDGF/p75 being the major lentiviral tethering protein used the IN-binding domain containing C-terminal portion of LEDGF/p75 fused to an alternate N-terminal domain displaying distinct DNA or chromatin binding preferences [121–124]. This was exemplified first *in vitro* using λR-LEDGF/p75 fusion proteins [121] and was recently confirmed *in vivo* as well [122–124]. Indeed, using LANA31 or histone 1-LEDGF/p75 chimeric proteins, Meehan *et al.* showed that they could rescue infectivity in LEDGF/p75 depleted cells [122]. Furthermore, the fusion of LEDGF/p75 to heterochromatin protein 1α (CBX5) [123] or heterochromatin protein 1β (CBX1) [124] altered HIV integration site selection, redirecting integration preferences from active transcription units to heterochromatin regions, thereby giving final proof for the tethering role of LEDGF/p75. Interestingly, these studies demonstrated that integration targeting site preferences may be modified *in vivo*, being of potential interest for improving safety of retroviral-based gene therapy vectors.

Up to now, LEDGF/p75 is the only tethering protein described for lentiviruses. However, alternate cellular proteins are likely to play a role in lentiviral PIC tethering as well. Foamy virus integration involved Gag tethering to H2A/H2B core histones [102]. Tethering proteins involved in integration of other retroviral genera are yet to be described.

To date, 13 proteins interacting with MLV IN have been identified by yeast two-hybrid and represent potential tethering protein candidates dictating MLV integration site selection preferences for promoter regions, as they are chromatin-binding proteins or transcription factors [125]. These candidates include transcription factors (TFIIE-β, B-ATF, Znfp15, Znfp38, Ankrd49, AF9), chromatin remodeling factors (brd2, Enx-1) and factors involved in DNA repair (Ku70, fen1). These putative candidates are consistent with an enrichment of transcription factor binding sites surrounding MLV integration sites [73]. Interestingly, some of the MLV IN interacting proteins (AF9, brd2, Znfp38, Ku70 and fen1) also interact with HIV IN in yeast two-hybrid, suggesting that they might play a role in HIV integration targeting in promoters in the absence of LEDGF/p75 [125].

#### 3.3.3. Epigenetic modifications and integration targeting

The Encyclopedia of DNA Elements (ENCODE) contains ∼1% of the genome that is extensively annotated, allowing comparisons of epigenetic marks with retroviral integration site preferences [126,127]. Consistent with previous findings on favored insertion in active transcription units, lentiviral integration sites associated significantly with the epigenetic marks H3K4me, H3K36me, H3K9/K14Ac and H4Ac, histone modifications usually associated with transcriptionally active chromatin. In contrast, lentiviral integration sites were disfavored in regions containing DNA CpG methylation as well as H3K9me2/3, H3K27me2/3 and H3K79me3, epigenetic marks usually associated with repressed chromatin [72,76]. A similar association pattern was observed for alpharetroviruses, gammaretroviruses and HERV-K [88].

Recently, LEDGF/p75 was identified as a cellular partner of the menin/MLL complex [128]. The menin/MLL histone methyltransferase complex promotes specific trimethylation of histone 3 on lysine 4 (H3K4me3), an epigenetic mark associated with active transcription. This histone modification is also associated with HIV integration sites, coherent with a global picture in which, LEDGF/p75, epigenetic marks associated with transcriptional activity and HIV integration sites converge to similar genomic locations.

## 4. Conclusions

LEDGF/p75 was initially identified in a complex co-immunoprecipating with positive cofactor 4 (PC4), a general coactivator of transcription [113,129]. PC4 has been involved in many transcription steps: (i) PC4, by interacting with upstream activators and the general transcriptional machinery, can enhance the efficiency of pre-initiation complex assembly, thereby promoting transcription initiation, in cooperation with TBP-associated factors (TAFs) [130]; (ii) PC4 improves transcription activation by stimulating promoter escape [131]; (iii) Sub1, the yeast PC4 homolog, facilitates transcription elongation and may also prevent premature transcription termination [132]. Thus, being associated to a PC4-containing complex, itself associated with the transcription machinery, it is reasonable to hypothesize that LEDGF/p75 might be associated with the RNA polymerase II complex during elongation.

**Figure 2.**
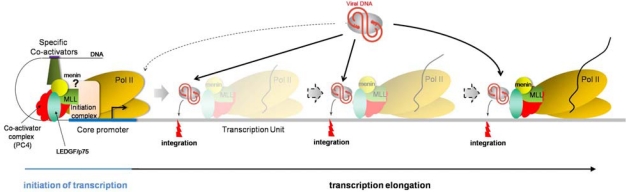
Dynamic model depicting the mechanism of LEDGF/p75-mediated HIV integration. LEDGF/p75 (green oval) associates with PC4 (red protein) and the RNA polymerase II machinery (yellow ovals) at promoter regions, but steric hindrance may prevent successful recruitment of preintegration complexes (gray oval with viral DNA in red). In this proposed model, LEDGF/p75 remains associated with the RNA pol II transcription elongation complex, potentially interacting with PC4 and menin/MLL complex. While this complex displaces nucleosomes (not depicted) and unwinds DNA to allow RNA polymerization, LEDGF/p75 may recruit HIV PIC and promote integration. This model is consistent with LEDGF/p75-captured DNA sequences and HIV integration sites being present throughout the transcription unit, without specific DNA binding consensus motif.

Therefore, based on the current knowledge about HIV integration site selection, *i.e.*, preferentially integrating into active transcription units, it is tempting to speculate a dynamic, more than a static, tethering model, in which LEDGF/p75 would be associated with PC4 and the RNA polymerase II elongation complex (Figure 2). In this model, LEDGF/p75 would recruit HIV preintegration complex while transcribing genes or at pausing sites, thereby explaining integration sites all along the transcription units.

Consistent with these results are (i) integration in active transcription units, with no preference along the transcription unit, neither for exons, nor for introns, (ii) LEDGF/p75 initially characterized as a transcriptional coactivator, associated with the transcription machinery (via PC4-containing complex), (iii) LEDGF/p75 interaction with the menin/MLL histone methyltransferase, involved in H3K4me3 histone modification, a mark associated with active transcription, and (iv) no sequence consensus for LEDGF/p75-binding DNA sites, suggesting that the N-terminal domain of LEDGF/p75 may serve as a hook to anchor the protein to the chromatin but that the location specificity is given by an additional chromosome-bound protein, yet to be identified.

Further studies on LEDGF/p75 should help refining the detailed mechanism of LEDGF/p75-mediated HIV integration.

## Figures and Tables

**Table 1.** Major genome-wide studies of retroviral integration distribution.

**Retroviridae genera**	**Specimen ^a^**	**Host cell type ^b^**	**Approx. Nb. of sites investigated ^c^**	**References**
**lentiviruses**	HIV-1	human	59869	[23,36,59–76]
		other	2421	[37,77–81]
	HIV-2	human	202	[82]
	SIV	human	148	[83]
		simian	501	[84]
	EIAV	human	1241	[69,81]
		other	70	[81]
	FIV	human	226	[85]
**alpharetroviruses**	ASLV	human	695	[62,86]
		avian	658	[77]
**betaretroviruses**	MMTV	human	298	[87]
		murine	170	[87]
**gammaretroviruses**	MLV	human	4005	[60,66,70,73,88]
		murine	189	[37]
		other	953	[78,79,84]
	MSCV	murine	259	[89]
	PERV	human	1962	[90,91]
	XMRV	human	472	[92]
**deltaretroviruses**	HTLV-I	human	1235	[93–95]
**epsilonretroviruses**	Not investigated			
**spumaviruses**	FV	human	3457	[65,70]
		other	263	[78]
**Endogenous retroviruses**	HERV-K	human	1565	[88]

a HIV: human immunodeficiency virus; SIV: simian immunodeficiency virus; EIAV: equine infectious anemia virus; FIV: feline immunodeficiency virus; ASLV: avian sarcoma leukosis virus; MMTV: mouse mammary tumor virus; MLV: murine leukemia virus; MSCV: murine stem cell virus; PERV: porcine endogenous retrovirus; XMRV: xenotropic murine leukemia virus-related virus; HTLV: human T-cell lymphotropic virus; FV: foamy virus; HERV: human endogenous retrovirus.

b Host cell type includes human, simian, murine, canine and avian cells. Are indicated the human cells and the host cell type specific to each specimen. Other: non human and non species-specific host cell type.

c Number of integration sites analyzed in untreated/control cells according to the original publication.

**Table 2.** Chromosomal features associated with preferential retroviral integration sites.

**Retroviridae genera**	**in Transcription Units^a^**	**± 2kb Transcription Start Sites^a^**	**± 2 kb CpG Islands^a^**
Lentiviruses	+/++	0	−/0
Alpharetroviruses	+	0	+
Betaretroviruses	0	0	0
Gammaretroviruses	+	++	++
Deltaretroviruses	+	+	+
Epsilonretroviruses	NA	NA	NA
Spumaviruses	0	++	++
HERV-class II	+	++	++

a ratio between the proportion of the chromosomal feature over the random proportion in the human genome, according to RefSeq databases and with values from [69,87,88,90,94,95].

0: no statistical difference over random;

+/++: statistically favored feature over random with ++ for ratio >2 and + for a ratio <2;

−: statistically disfavored feature over random

NA : not available
